# Composite variable bias: causal analysis of weight outcomes

**DOI:** 10.1038/s41366-025-01732-6

**Published:** 2025-03-08

**Authors:** Ridda Ali, Andrew Prestwich, Jiaqi Ge, Claire Griffiths, Richard Allmendinger, Azar Shahgholian, Yu-wang Chen, Mohammad Ali Mansournia, Mark S. Gilthorpe

**Affiliations:** 1 Alan Turing Institute, London, UK; 2 Leeds Institute for Data Analytics, University of Leeds, Leeds, UK; 3 School of Geography, University of Leeds, Leeds, UK; 4 School of Psychology, University of Leeds, Leeds, UK; 5 Obesity Institute, Leeds Beckett University, Leeds, UK; 6 Alliance Manchester Business School, The University of Manchester, Manchester, UK; 7 Liverpool Business School, Liverpool John Moores University, Liverpool, UK; 8 Department of Epidemiology and Biostatistics, School of Public Health, Tehran University of Medical Sciences, Tehran, Iran

**Keywords:** Epidemiology, Public health, Health policy, Weight management

## Abstract

**Background:**

Researchers often use composite variables (e.g., BMI and change scores). By combining multiple variables (e.g., height and weight or follow-up weight and baseline weight) into a single variable it becomes challenging to untangle the causal roles of each component variable. Composite variable bias—an issue previously identified for exposure variables that may yield misleading causal inferences—is illustrated as a similar concern for composite outcomes. We explain how this occurs for composite weight outcomes: BMI, ‘weight change’, their combination ‘BMI change’, and variations involving relative change.

**Methods:**

Data from the National Child Development Study (NCDS) cohort surveys (*n* = 9223) were analysed to estimate the causal effect of ethnicity, sex, economic status, malaise score, and baseline height/weight at age 23 on weight-related outcomes at age 33. The analyses were informed by a directed acyclic graph (DAG) to demonstrate the extent of composite variable bias for various weight outcomes.

**Results:**

Estimated causal effects differed across different weight outcomes. The analyses of follow-up BMI, ‘weight change’, ‘BMI change’, or relative change in body size yielded results that could lead to potentially different inferences for an intervention.

**Conclusions:**

This is the first study to illustrate that causal estimates on composite weight outcomes vary and can lead to potentially misleading inferences. It is recommended that only follow-up weight be analysed while conditioning on baseline weight for meaningful estimates. How conditioning on baseline weight is implemented depends on whether baseline weight precedes or follows the exposure of interest. For the former, conditioning on baseline weight may be achieved by inclusion in the regression model or via a propensity score. For the latter, alternative strategies are necessary to model the joint effects of the exposure and baseline weight—the choice of strategy can be informed by a DAG.

## Introduction

Observational research frequently involves composite variables—i.e., algebraically derived variables that are created by adding, subtracting, multiplying, or dividing two or more distinct variables [1]. In obesity research, a common study outcome, body mass index (BMI), is an inherently composite variable that incorporates both height and weight components and is derived by dividing body weight (in kilograms) by height (in metres) squared. Researchers in the field of obesity can also construct composite variables such as changes in body weight or size (by subtracting baseline weight or size from follow-up weight or size) [2–6], or relative change in body weight or size (a more convoluted ratio variable construct that derives a ratio measure of a weight or size change-score with respect to the baseline outcome value) [7]. While change scores such as weight change or relative weight change may not always be viewed by applied researchers as composite variables in the same way as BMI, they are nevertheless composite because, in these cases, they are derived by the subtraction or division of two or more distinct variables.

In observational studies, including in the field of obesity research where researchers wish to identify factors that contribute to weight gain or weight loss, causal inferences are often sought. However, most observational studies fail to employ appropriate causal inference methods. Where causal inference methods are deployed, it is necessary to treat composite variables very carefully, including those that are either inherently composite (such as BMI) or constructed to be composite (such as weight change or relative change). The goal of this article is to highlight issues regarding the use of such composite variables—a concern for obesity researchers given the widespread use of measures such as BMI and weight change—as well as recommending alternative approaches to using BMI or weight change scores as outcomes.

It has been recognised previously that analysing composites as an *exposure* is problematic, with study estimates suffering *composite variable bias* [1]; we extend this to scenarios where composite variables are analysed as *outcomes*. The problem is *inferential bias* made by the researcher in what they anticipate to be true, when in reality there is a mismatch between what is asked (estimand) and what is answered (estimate) because the statistical machinery deployed for the composite outcome returns the wrong answer [8].

Many concerns over the use of change scores (such as weight change from baseline to follow-up) and/or conditioning on baseline outcomes (such as weight) have been made in the statistical literature that are often ignored [9, 10]—perhaps because the statistical literature is less accessible or less well read. We offer an alternative way to understand these problems using directed acyclic graphs (DAGs), which are based on complex mathematical theory that has undergone rigorous development and thorough evaluation within the technical literature of statistics, mathematics, and computer science [11]. Many powerful algorithms have been developed using probabilistic graphical models, enabling a DAG to identify which statistical process delivers robust causal insight without the need to be an expert in graph theory [12]. Since problems with composite *exposures* have been discussed already [1], we examine the causal effect of multiple variables on composite *outcomes* using a DAG to illustrate how different analyses are needed to answer different causal questions, and explain why estimands involving a composite outcome cannot be reliably estimated. We examine the phenomenon of composite variable bias for outcome measures of body weight and BMI, although the same issues apply to other measures of body size (such as waist-to-hip ratio, WHT; or the body roundness index, BRI).

We might for instance ask how factors such as diet, physical activity, and mental health influence body weight over time—perfectly legitimate causal questions (estimands). To estimate our estimand we must ensure that the quantification (*causal effect*) is accurate, i.e., if we intervene on a factor, we know by how much body weight is (on average) affected. When examining changes in body weight, we might proceed in many ways; for instance, we may calculate a change-score of weight (follow-up minus baseline) and use that as an outcome; alternatively, we might use body mass index (BMI) and could similarly construct a change-score outcome for BMI (follow-up BMI minus baseline BMI); or we might prefer a relative measure of change in body weight (relative change in either weight or BMI relative to baseline weight or baseline BMI). All of these introduce methodological issues that arise because the outcome is composite.

The issue with any composite, whether change-score (follow-up minus baseline), ratio (BMI) or other fraction (relative change), is that composite values have a one-to-many relationship with their components: i.e., the same change-score corresponds to an infinite combination of follow-up and baseline values; the same BMI corresponds to an infinite combination of weight and height values; and the same relative change corresponds to an infinite combination of baseline and follow-up values. It is then impossible to unpick the separate causal relationships associated with each component, and the causal effect estimated may no longer correspond to changes observed were an intervention undertaken. This study outlines why and relates this to examples in the obesity research literature that examine body weight *changes* over the study period.

Change scores conflate two measures at a single time point [1, 8, 13]. This can yield misleading causal inferences that distort the causal effect estimated. The exposure may be related to the baseline outcome as well as the follow-up outcome, making it challenging to unpick the causal relationships that occur for both outcome measures simultaneously. BMI also has two components that stabilise (i.e., when values no longer vary due to growth) at different times—height stabilises around the end of adolescence, whereas weight may continue to alter and stabilise for shorter periods around when measured. BMI conflates the separate causal relationships associated with height and weight, making it difficult to unpick the causal relationship for just weight (Supplementary Section 1). In all instances, the one-to-many correspondence of composite values and the multiple possible values of all components is the root cause of inferential biases that emerge. This is more pronounced in obesity research because outcomes are often built around BMI and/or changes in BMI, and samples with near-identical BMI distributions may possess different joint distributions of height and weight due to differences in sample composition by sex, ethnicity, or any trait associated with height or weight. Adjustment for sex and ethnicity or any trait associated with height or weight may then further confuse the interpretation of average causal effect of an exposure on BMI. In general, reliable analytical approaches need to focus on the individual components of a composite outcome to avoid misleading causal inferences.

There are also problems when an exposure precedes baseline assessments of body weight and interest remains in the assessment of this exposure in relation to body weight *changes*. For instance, Katsoulis et al. [14] sought to identify adult individuals who are more likely to gain weight by using Electronic Health Records (EHR) to assess the relationship between changes in BMI and demographic factors such as age, sex, and ethnicity. Their findings suggested that the youngest age range (18–24 years) were more prone to weight gain in comparison to those in the oldest age group (65–74 years). However, birth variables (e.g., sex) can only be evaluated as exposures for follow-up weight and not *changes* in weight (or *changes* in BMI), since baseline weight (part of baseline BMI) mediates their exposure.

To assess the impact on weight *change*, it remains necessary to model follow-up weight conditioned on baseline weight, but it is inappropriate to include baseline weight in the regression analysis (either directly or via a propensity score) [15], because this invokes conditioning on baseline weight as a mediator. This alters the causal effect estimate from *total* to *direct* effect (Supplementary Section 2) [16] and risks collider bias, which arises from inappropriate conditioning on a mediator downstream of the exposure (a variable that causes the outcome but is also caused by the exposure and many other unknown causes of the outcome) [17–20]. As noted half a century ago, ‘*prognostic variables should not themselves be influenced by treatment, otherwise in correcting for differences in prognostic variables one may unwittingly remove some of the treatment effect*’ [21]. This is why alternative approaches to examine birth variables in relation to weight *change* must be considered; this may involve causal mediation analysis [22–27], which determines the *joint* causal effects of the birth variable and baseline weight in a manner that remains reliable for causal effect estimation. It is still viable to examine birth variables in relation to follow-up weight only, but this does not answer any questions in relation to weight *change*.

It is worth noting that some weight-loss studies examine *weight change* in relation to *baseline weight* to evaluate the hypothesis that heavier individuals at the start of a study lose more weight than lighter individuals [28, 29]. Such analyses are also problematic because this type of analysis invokes a mathematical tautology, where the baseline measure of interest is on both sides of the regression equation, leading to misleading causal inferences [29, 30]. Studies also investigate time-varying outcomes, time-varying exposures, and time-varying confounding—such situations warrant the use of g-methods [31].

All studies outlined illustrate what is typically observed across the literature for many investigations into body weight and factors that might affect their *changes*. Studies that seek causal insights using composite body weight measures as outcomes are ubiquitous, yet few yield reliable causal insights due to methodological oversights. Where these studies are used to inform decisions made by health practitioners and/or policymakers [32–34], there is potential for misguided decisions informed by a misleading evidence base.

## Methods

### Illustrating these issues through a causal perspective

To illustrate the inferential bias that arises from incorrect application of statistical machinery to the analysis of composite outcomes, we identified candidate datasets in the public domain that had the following characteristics (inclusion criteria): (1) longitudinal study design to obtain baseline and follow-up body size measures; (2) comprised adults, as heights would have stabilised in early adulthood (i.e., no more growth, which might lead researchers to believe that BMI comprises only weight changes *within* individuals, whereas methods used contrast height differences *between* individuals—Supplementary Section 3); (3) baseline measures (e.g., sex, ethnicity) as confounders for key exposures of interest; (4) baseline height and weight, for confounding of the key exposures and to create composite outcomes when combined with follow-up measures (e.g., change in BMI); (5) at least one suitable ‘exposure’ (i.e., whose causal effect is to be estimated) that could causally impact weight changes over time; (6) follow-up height and weight to generate composite outcomes; and (7) <10% missingness, as statistical analyses are likely biased with more missingness [35].

Of the 18 datasets accessed through the UK Data Service, only the National Child Development Study (NCDS) cohort surveys at ages 23 and 33 met most inclusion criteria, though missingness due to loss-to-follow-up at age 23 remained a problem. What follows is therefore illustrative only (see Supplementary Table S1 for datasets considered, reasons for exclusion, and discussion around loss-to-follow-up). The NCDS documented the lives of children who were born in Britain during a week in 1958 [36]. Heights and weights of cohort members were self-reported (with potential for inaccuracies and bias) at age 23, whereas at age 33 they were measured by medical staff [37]. There are thus two measures for height across this 10-year period (Supplementary Section 4). Table 1 defines all variables from the NCDS surveys used for our analyses. Supplementary Fig. S1 shows the participant flow chart. A more detailed overview of the dataset (summary statistics, frequency, etc.) are provided in Supplementary Fig. S2.

**Table 1:** Data summary (variables with their definitions).

Variable	Definition
Ethnicity	Value: 0 (White) Value: 1 (Non-white)
Sex	Value: 0 (Female) Value: 1 (Male)
Baseline height	Height at age 23
Baseline weight	Weight at age 23
Baseline BMI	BMI at age 23
Economic status (measured at age 23)	Value: 0 (Full-time education or employed) Value: 1 (Unemployed or economically inactive)
Malaise score (dichotomised by the study and measured at age 23)	Value: 1 (Normal = score 0–7) Value: 0 (Depressed = score 8–24)
Follow-up weight	Weight at age 33
Follow-up height	Height at age 33
Follow-up BMI	BMI at age 33
Change in BMI	Follow-up BMI − Baseline BMI
Relative change in BMI	(Follow-up BMI − Baseline BMI) $\div$ Baseline BMI
Change in weight	Follow-up weight − Baseline weight
Relative change in weight	(Follow-up weight − Baseline weight) $\div$ Baseline weight

### DAG-informed analyses

A directed acyclic graph (DAG) is a causal path diagram based on graph theory [12] used to visually encode hypothesised or established causal relationships among variables [38–44]. When estimating causal effects, DAGs provide the identification of confounders that causally precede both the exposure and outcome, and which should be included in the statistical model directly (or via propensity scores) to minimise confounding bias. DAGs also identify which variables are mediators (i.e., that occur *after* the exposure, except for the outcome variable) which should not be included in the model, as this introduces collider bias [19, 21, 38–43]. Observed variables are depicted in squares or rectangles in the DAG while unobserved (latent) variables are depicted in circles or ellipses. Each variable in a DAG is assumed to be a possible cause of all future variables except for variables involved in fully deterministic relationships (e.g., BMI is fully determined by height and weight), or where there is a theoretical basis or convincing evidence of no causal relationships.

**Fig. 1:**
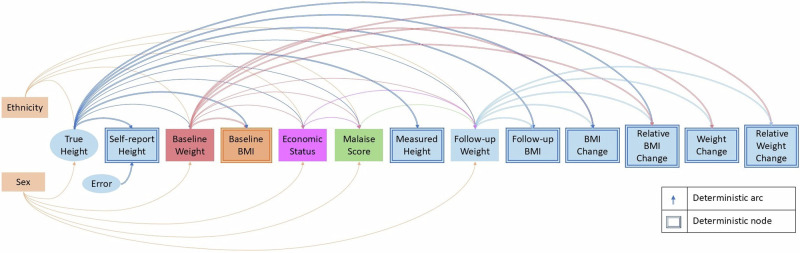
Directed acyclic graph (DAG) illustrating the temporal order of the variables. Observed variables are depicted in squares or rectangles while unobserved (latent) variables are depicted in circles or ellipses. The socioeconomic (economic status) and psychological (malaise score) variables in the NCDS data are composite measures of a latent construct that are treated as observed variables; they lie between height/weight at baseline and weight at follow-up. All other composite variables are fully determined by other variables and denoted by double-outlined nodes. True unmeasured height occurs at age 23, when observed as self-reported height, including potential unobserved errors, while height at age 33 is the error-free measured height identical to unobserved true height at age 23.

Figure 1 shows the DAG codifying how variables were assumed to be causally associated with each other in the NCDS data, with outcomes often considered in observational studies denoted in light blue (e.g., follow-up weight). The order of the variables and the direction of the arrows were determined by the temporal order in which variables stabilised. Ethnicity and sex stabilised at birth, and baseline height and baseline weight were used to calculate the fully determined baseline BMI, hence they preceded this composite variable. From the perspective of post-adolescence, weight *change* temporarily follows height stabilisation, hence we chose height to causally precede weight within adults. Although true height at age 33 is likely identical to true height at age 23, height was self-reported at age 23 while measured by researchers at age 33. We treated measured height as true height, though in many instances measured height may contain measurement error. For the purposes of our study, we treated self-reported height as the fully determined combination of unobserved true height and unobserved self-report error. This distinction was necessary because of discrepancies between self-reported and measured heights—we adopted measured height as the reliable (i.e., ‘true’) value for height at both age 23 and age 33.

Malaise score was obtained from a self-administered questionnaire that participants completed at age 23 to assess psychiatric morbidity (e.g., depression) at that time, and, in the DAG, this occurs after economic status (also measured at age 23) since early adult economic status stabilised earlier and over a longer period than did malaise score—psychological status is likely more transient than economic status over the lifecourse. Both exposure variables are composite but capture a latent measure. It is therefore not necessary to deconstruct these measures, and they are treated as observed variables. All other composite variables are fully determined by other variables and denoted by double-outlined nodes; no other variables have arcs into a fully determined variable (e.g., ethnicity may cause height and weight but not BMI); and there are no forward arcs from any fully determined variable, as their causal contribution is captured by the variables that determine them [1].

Various exposures of interest are denoted in different colours to clearly show the arrows coming out of them. All variables in different colours are potential exposures of interest and a different model maybe needed for each exposure of interest, informed by the DAG [16]. This is illustrated as we examine the causal role of each potential exposure variable in the DAG.

Multiple DAG-informed linear regression models were generated to explore the role of each variable for its causal impact on body size at age 33. Depending on where each variable sits in relation to baseline measure of body size at age 23, the exposure either impacts *follow-up* body size (at age 33) or *change* in body size (from age 23 to age 33). For example, to estimate the causal effect of economic status (at age 23) on follow-up weight, we condition on ethnicity (determined at birth), sex (determined at birth), baseline height (age 23), and baseline weight (age 23) because these are the indicated confounders for the causal relationship examined (e.g., ethnicity is a potential common cause of both economic status and follow-up weight). We do not condition on the malaise score as it is a mediator (i.e., malaise score is on the causal path between economic status and follow-up weight; Supplementary Fig. S3). Adjusting for baseline weight is essential to seek the causal effects of an exposure on *change* in weight, because we must condition on baseline weight to remove the component of follow-up weight that is not baseline weight. Adjusting for baseline weight alters the interpretation of the causal effect estimated—a mathematical exposition of this is provided in Supplementary Section 5.

Causal questions about birth variables (that precede baseline weight), such as ethnicity and sex, can only be evaluated with respect to follow-up weight and not weight change because adjusting for baseline weight involves adjusting for a mediator. To ask causal questions about birth variables in relation to *weight change* it is necessary to use alternative strategies, such as mediation analysis [22–27], which determines the *joint* causal effects of birth variable(s) and baseline weight. Such strategies are beyond the scope of this study, but a brief description of mediation analysis is provided in Supplementary Section 2.

**Fig. 2:**
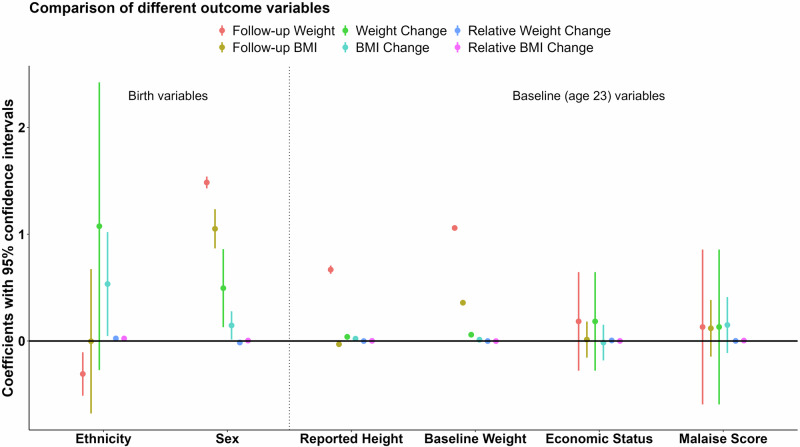
A comparison of the results for different weight outcomes for different exposure variables of interest—the dotted vertical line separates the birth variables from the baseline (age 23) variables (see Supplementary Table S2 for explanation of differences in causal interpretation). The effect of ethnicity and sex differences on follow-up weight were ten-fold greater than the coefficients shown in the plot and were rescaled to make the plot more readable.

Inevitably, analyses of different outcomes are anticipated to give different estimates, but the only outcome analysis that is interpretable from a causal perspective is that for follow-up weight. For exposures after baseline weight their causal impact on weight *change* is estimated by adjusting for baseline weight. For exposures before baseline weight (i.e., birth variables) their causal impact on *follow-up* weight only is estimated. Alternative outcome analyses—BMI, (relative) weight changes, (relative) BMI changes—are all composites and yield inferences that cannot be interpreted causally. We illustrate these outcome analyses because they are so often considered in the obesity literature. DAG-informed linear models were generated for six weight outcomes (follow-up weight, follow-up BMI, BMI change, relative BMI change, weight change, and relative weight change) to show the effect sizes for the reliable (follow-up weight) and misleading (e.g., follow-up BMI) analyses, summarised visually in Fig. 2 (with detailed results provided in Supplementary Table S3).

All analyses were conducted using the ${lm}$ regression package in R (version 4.4.2; R Development Core Team) using the RStudio (version 2024.09.1) interface platform (all the code is publicly available at https://github.com/RiddaAli/Composite-Variable-Bias-IJO.git).

## Results

It is important to note that we are interested in causal effect sizes for clinical significance, not statistical significance, as it is the magnitude of purported change in the outcome for a given change in the exposure that we seek.

### Birth variables

Follow-up weight (kilogram) was lower in the Non-White group compared to the White group (−3.088; 95% confidence interval (CI), −5.121 to −1.054). Follow-up BMI showed no substantial difference between White and Non-White groups (−0.002; 95% CI, −0.679 to 0.674). The remaining four outcome measures (weight change (1.075; 95% CI, −0.273 to 2.423), BMI change (0.534; 95% CI, 0.047 to 1.021), relative weight change (0.024; 95% CI, 0.004 to 0.044) and relative BMI change (0.023; 95% CI, 0.002 to 0.045)) were higher in the Non-White group than the White group. The composite nature of follow-up BMI, relative weight change, and relative BMI yielded estimates close to the null and all other outcomes yielded estimates that were sign-reversed from the only reliable causal effect estimate (i.e., follow-up weight as the outcome)—this reflects how follow-up BMI is different from follow-up weight because BMI conflates weight and height, with the latter differing by ethnic background (i.e., the white group is taller than non-white group).

Males were substantially heavier than females at follow-up (14.851; 95% CI 14.299 to 15.403), which is not surprising given how mean body size differences between males and females begin at birth and this population-level sex difference is maintained throughout life. The estimated sex difference was diluted for follow-up BMI as the outcome. When change scores or relative change measures were analysed, estimates were smaller or close to zero for BMI change and relative BMI change, and sign-reversed for relative weight change (−0.014; 95% CI, −0.020 to −0.009) compared to all other estimates.

For later exposures (baseline height, baseline weight, economic status, and malaise score), ethnicity and sex are confounders and their model parameter estimates cannot be interpreted [16].

### Baseline height and weight

It may seem of little interest to examine the estimated causal impact of baseline height and baseline weight on various weight outcomes, yet the analysis of weight change with respect to baseline weight is sometimes erroneously considered a viable research question [28].

The causal effect of height on weight at follow-up was unsurprisingly positive, revealing its adjustment as a confounder to be important. Self-reported height (age 23) and measured height (age 33) were two versions of the same baseline variable and each caused weight at follow-up (self-report: one centimetre more reported baseline height corresponded to 0.668 (95% CI, 0.630 to 0.707) kilograms more weight at follow-up; measured: one centimetre more measured baseline height corresponded to 0.694 (95% CI, 0.655 to 0.734) kilograms more weight at follow-up), whereas similar estimates were either diluted for all the other outcomes or sign-reversed, and sensitivity analysis revealed that small differences were found if measured height was used in place of self-reported height.

Unsurprisingly, baseline weight (adjusted for self-reported baseline height) was related to follow-up weight (one kilogram more baseline weight resulted in 1.059 (95% CI, 1.038 to 1.080) kilograms more weight at follow-up). The estimated causal effect of weight on BMI was substantial (one kilogram more baseline weight resulted in 0.359 (95% CI, 0.351 to 0.366) higher BMI at follow-up), which is expected given that BMI comprises weight. Estimates for the causal impact of baseline weight on all other weight outcomes were close to zero. Sensitivity analysis again revealed small differences if adjustment was made instead for measured height.

### Economic status and malaise score

When examining economic status and malaise score as key exposures for their impact on follow-up weight, baseline weight is a confounder, meaning causal evaluation was in terms of their impact on *weight change*. Economic status and malaise score are binary variables, providing less information than a more granular continuous measure, leading to effect dilution due to dichotomisation [45]. In the NCDS data, the effect sizes of both binary measures were small, yet their analyses revealed subtle differences for different weight outcomes.

It is important to note that change score models (e.g., BMI change as the outcome) and follow-up models (e.g., follow-up BMI as the outcome) are mathematically equivalent when adjusting for the baseline outcome variable (e.g., baseline BMI; more examples are provided in Supplementary Table S4 and Supplementary Section 6). This only illustrates how similar (or identical) estimates may arise, but where only one has theoretical underpinnings from a causal perspective—it is knowledge (or theory) not present in the data but encoded in the DAG that informs which analysis reliably estimates a causal effect.

We did not adjust for the baseline composite variable (baseline BMI) when analysing the BMI change and follow-up BMI outcomes (as informed by the DAG containing these variables as fully determined with no onward arcs); we instead adjusted for baseline height and baseline weight (as informed by the DAG). Therefore, the estimates for these outcomes are not mathematically equivalent. However, the estimates for follow-up weight and weight change outcomes are mathematically equivalent for malaise score and economic status because we did adjust for baseline weight (as informed by the DAG).

The effect of economic status on follow-up weight and weight change (adjusting for baseline weight and self-reported height) was slightly higher than all other weight outcomes. The effect of malaise score on follow-up weight and weight change was slightly lower than all other weight outcomes. Sensitivity analysis revealed that using measured height instead of self-reported height yielded slightly different results (Supplementary Fig. S4), indicating the importance of correctly measuring height as a confounder. Additionally, not adjusting for baseline height and baseline weight resulted in larger differences (even sign reversal) for the various weight outcomes (Supplementary Fig. S5). For the only meaningful causal estimates (i.e., follow-up weight as the outcome while conditioning on baseline weight) we observed no sizeable effect estimates for either key exposure on change in weight.

## Discussion

Findings show that model estimates differ across outcomes to the point where different conclusions might be drawn for each outcome, even when the outcomes seem conceptually relatively similar (e.g., BMI change and weight change).

To understand causal relationships involving *weight change*, analyses should use follow-up weight adjusting for baseline weight (using appropriate causal inference methods) rather than follow-up BMI, weight change, BMI change, or any other composite measure of body size and body size changes. The necessity to examine *change* this way is indicated by graphical model theory that underpins DAGs, since the DAG can only indicate variables to be conditioned on (for confounding adjustment) if they are probabilistic (not deterministic) else the underpinning graphical model theory cannot work [1]. Further, to analyse outcome *change*, it is necessary to isolate all aspects of the outcome follow-up measure that is *not* determined by its baseline measure, which can only be achieved by conditioning follow-up on baseline [8]. Where attempts are made to draw meaningful inferences from composite outcomes, most model estimates will not quantify cause and effect, and estimates may not relate to observed consequences in the outcome following an intervention on the exposure.

BMI may be useful for describing a population, but it cannot provide reliable causal insights at the individual level. Many consider BMI to be a proxy for adiposity (Supplementary Section 1), but it is worth noting that there is no height-invariant measure of body composition; and attempts to ‘standardise’ weight through dividing it by height squared cannot achieve a height-invariant measure. This only leads to BMI variance being on average two thirds height and one third weight for most adult populations. The statistical challenges with constructed ratio variables have been extensively discussed many times since the inception of modern statistics [46–48]. The only reliable way to examine weight as if it were height-invariant is to condition on height in the modelling process, as illustrated in this study.

Statistical adjustment for confounding can be challenging when the composite outcome spans a period in which some important variables are both a confounder for one parent of the composite (e.g., adulthood diet confounds weight for the outcome BMI) while also a mediator of another parent of the composite (e.g., adulthood diet mediates height for the outcome BMI)—this prohibits statistical adjustment and is sufficient reason to deconstruct composite variables and analyse their distinct component variables.

If baseline weight precedes the exposure of interest, investigation of the impact of an exposure on weight *changes* is achieved by conditioning on baseline weight—either directly including it in the model or via propensity scores [15]. If baseline weight follows the exposure of interest, conditioning on baseline weight is still required, but this will involve more complex analytical strategies [22–27]. The choice of method may be informed by the development of an appropriate DAG. DAGs are useful to consider deterministic variables and their components [1], to identify and avoid issues in the analysis of composite variables (as outcomes and/or exposures), indicating which strategies yield reliable causal effect estimates for the components of each composite (since graph theory underpinning DAGs does not work for deterministic variables). Analyses of follow-up outcomes conditioned on baseline outcomes nevertheless require careful consideration to obtain valid standard errors [49, 50].

Occasionally, estimated coefficients may appear similar or even identical, prompting the question, is our worry about composite outcomes a major concern? The key issue is that, irrespective of whether estimates for different analytical strategies are similar or not, the only analysis that is generally reliable is that informed by external knowledge (or theory) as encoded in a DAG. It then emerges that the only generally reliable causal effect estimate is that for follow-up weight as the outcome while conditioning on baseline weight. All other strategies of analysis may mislead in some or most other instances, and the occurrence or extent of this is generally unknown. Where reliable causal inference approaches are not considered, research practices risk generating misleading findings that may lead to erroneous policy decisions.

Although Stevens et al. [51] discuss inconsistencies in the definition of weight maintenance across studies, stating that percentage change in BMI is the same as percentage change in weight in adults because their height is stable—this is inaccurate as this is only true for within-person analyses. The regression methods deployed make both within- and between-person contrasts (Supplementary Section 3). It is thus incorrect to assume that examining BMI is equivalent to examining weight among individuals with stable height for most contexts and most analyses. Intriguingly, we demonstrated that when presented with two assessments of (stabilised) height (self-reported versus measured by the research team), it is possible to use either, but differences in causal effect estimates arose. This shows how measurement error and/or self-report bias was likely present for the self-reported measure.

It is worth emphasising that the same analytical challenges arise for *all* composite outcomes and issues arise if we use composite body size measures as exposures [52], since regression methods make both within- and between-person contrasts for the exposure as well as the outcome. In general, analysis of composite measures as either exposure or outcome (except for proxy measures of latent variables) runs the risk of estimating misleading estimates of causal effect. We should be wary of all composite measures and question the utility of new ones. For instance, Wu et al. [53] recently created a new anthropometric index—the *body roundness index* (BRI)—which seeks to capture the percentages of total and regional fat by merging height and waist circumference. The authors concluded that BRI trends as an exposure were linked to an increased probability of developing cardiovascular disease, particularly among adults who were younger. Similarly, Krakauer and Krakauer [54] created *a body shape index* (ABSI) based on waist circumference adjusted for height and weight to assess abdominal adiposity. They concluded that ABSI as an exposure is a ‘risk factor’ for early mortality. Unfortunately, model estimates derived using BRI or ABSI as exposures or outcomes suffer composite variable bias and any causal estimates will not be reliable.

## Conclusion

Composite variables are commonly used in health research despite their interpretational challenges [1, 13, 29]. Although illustrated for body size outcomes used in obesity research, the issues raised here for composites as outcomes or exposures are applicable to all composites (e.g., GDP per capita, adverse childhood experiences, frailty index). BMI may be a useful indicator of health at the population level, but not at the individual level, and serves no reliable role in causal inquiry. Future studies aiming to estimate causal effects should avoid using composites as outcomes or exposures and instead use only non-derived variables. DAGs provide a useful strategy to identify and understand these issues [55, 56]. Thinking causally is essential to avoid erroneous conclusions and to meaningfully inform policy.

## Supplementary information

Supplementary Material

Supplementary Figure S1

Supplementary Figure S2

Supplementary Figure S3

Supplementary Figure S4

Supplementary Figure S5

## Data Availability

The data underlying this article can be downloaded from the UK Data Service. The analytic code is available at https://github.com/RiddaAli/Composite-Variable-Bias-IJO.git.
