# Supplementary material for: Composite variable bias: causal analysis of weight outcomes
Source: Int J Obes (Lond). 2025 Mar 8;49(6):1043–50. doi: 10.1038/s41366-025-01732-6 (PMC12158777; doi:10.1038/s41366-025-01732-6)
Supplement: Supplementary file 1 — Supplementary Material [file 41366_2025_1732_MOESM1_ESM.docx]

# Composite variable bias: causal analysis of weight outcomes

ᶲRidda Ali^1,2,3^, Andrew Prestwich^4^, Jiaqi Ge^1,2,3^, Claire Griffiths^5^, Richard Allmendinger^1,6^, Azar Shahgholian^7^, Yu-wang Chen^1,6^, *Mohammad Ali Mansournia^8^, *Mark S Gilthorpe^1,5^

1. Alan Turing Institute, London, UK.
2. Leeds Institute for Data Analytics, University of Leeds, Leeds, UK.
3. School of Geography, University of Leeds, Leeds, UK.
4. School of Psychology, University of Leeds, Leeds, UK.
5. Obesity Institute, Leeds Beckett University, Leeds, UK.
6. Alliance Manchester Business School, The University of Manchester, Manchester, UK.
7. Liverpool Business School, Liverpool John Moores University, Liverpool, UK.
8. Department of Epidemiology and Biostatistics, School of Public Health, Tehran University of Medical Sciences, Tehran, Iran.

## *Joint Senior / Corresponding Authors:

Prof Mark S Gilthorpe, Obesity Institute, Leeds Beckett University, Leeds, LS1 3HE, UK. Telephone number: (+44) 113 812 3160. Email: [m.s.gilthorpe@leedsbeckett.ac.uk](mailto:m.s.gilthorpe@leedsbeckett.ac.uk).

Prof Mohammad A Mansournia, Department of Epidemiology and Biostatistics, School of Public Health, Tehran University of Medical Sciences, Tehran, Iran. Telephone number: (+98) 21 8898 9123. Email: mansournia_ma@yahoo.com.

SUPPLEMENTARY MATERIAL

The following are the key take home messages, and the rest of the supplementary material addresses specific points and details within subsections.

- In the analysis of National Child Development Study (NCDS) cohort surveys at ages 23 and 33, estimates and associated inferences differed across different weight outcomes (e.g., BMI, weight change, etc.).
- Analyses of weight outcomes (e.g., BMI, weight change, etc.) are misleading from the perspective of causal inference if the outcome is composite – i.e., an unobserved constructed variable comprising multiple observed components – with potential sign reversal for different estimates derived from the same data.
- Although these analyses are explicitly different in mathematical terms (i.e., they are clearly different estimands), there may be a tendency to (incorrectly) assume they provide only a subtly different take on the same question: i.e., what is the effect of X on the change in some specified measure of body weight – the only robust estimands, however, are those with no composite outcome (or composite exposure).
- To understand causal relationships involving weight outcomes, analyses should use weight, not BMI, nor any other variation such as change-scores for either weight or BMI, nor percentage changes in either weight or BMI (the latter only makes the composite outcome more convoluted and impossible to untangle within a causal framework).
- To understand causal relationships involving change in weight outcomes, analyses should use follow-up weight (and none of the alternatives demonstrated here) and appropriately condition on baseline weight, as informed by a directed acyclic graph (DAG) – either treating baseline weight as a confounder or treating baseline weight as a joint exposure (e.g., within a causal mediation analysis) if it is a mediator of the exposure of interest.
- A key reason that composite outcomes (and composite exposures) are challenging to work with if seeking casual inquiry is that confounding adjustment cannot be formulated easily. The underpinning mathematics of DAGs (or potential outcomes) do not work for composite (fully determined) variables.

## Supplementary Section 1: Explanation of why BMI is not useful for causal understanding

For descriptive purposes, BMI and any other composite may be used with impunity, but within analyses that seek causal understanding, the estimation of a causal effect is challenging for any composite for the reasons outlined. In theory, it is possible to pose causal questions about BMI but answering them reliably and consistently is not possible – this is not a philosophical perspective, mere speculation, or opinion, but methodological fact because two causal effects (one pertaining to weight and one pertaining to height) are conflated and we currently have no methods that can unpick this effectively.

For BMI as an exposure there is consistency violation because for any given BMI value there are infinite combinations of height and weight that yield that value; and for BMI as the outcome, there is consistency violation because for any one sample of BMI outcome values there are an infinite number of alternative samples with the same BMI distribution where height and weight distributions differ. In both instances, causal effects cannot be interpreted reliably and consistently.

In practice, it is possible to place BMI in a DAG (i.e., treat BMI as a single variable and estimate the causal effect of X on BMI or the causal effect of BMI on Y), but due to conflating the causal relationships of two parent variables (height and weight), the relative contribution of each parent variable is weighted by its variance as depicted within the composite. BMI comprises a mixture of weight and height according to the variances of weight and height-squared in the sample. The conflation of variances means that, for most populations, the variance of height-squared is twice that of the variance of weight, meaning that BMI as an outcome or exposure will comprise two thirds (inverse) height squared and one third weight – and it is impossible to fully disentangle their causal roles. This also creates a challenge if seeking to make appropriate adjustments for potential confounding because there is currently no robust method to identify what confounders should be conditioned on.

For individuals over a certain age, their height might be constant well into older age, but regression methods estimate the necessary counterfactuals by making between-person contrasts within the data. For a single BMI value, contrasts within an observational dataset correspond to an infinite number of hypothetical people with weight and height values that yield the same BMI. There is no consistent causal effect to be estimated for a specific BMI as either an exposure or outcome because of this.

Weight scaled to height does not “standardise” weight for height, it conflates one over height-squared with weight, whereas weight conditioned on height correctly removes the confounding of varying individual heights within the population.

## Supplementary Section 2: Explanation of why alternative strategies such as Causal Mediation Analysing (CMA) are beyond the scope of this paper

It would require a separate paper to explain and illustrate the full complexities of approaches like CMA, which is why we outline the principles of this methodology only in brief. CMA examines jointly the primary causal exposure of interest (for which we seek to estimate its causal effect on the outcome of change in an outcome, i.e., the follow-up outcome conditioned on the baseline outcome, to isolate the causal impact of the exposure on the change part) and the baseline outcome as an additional exposure. This is done simultaneously in a single regression model. It is not feasible to use the standard form of regression analysis, where adjustment for the baseline outcome is adjusting for a mediator, as this alters the causal estimand to be estimating the direct causal effect of the exposure and not its total causal effect (which is what is being sought in most instances). A thorough examination of mediation analysis has been reported elsewhere [1].

## Supplementary Section 3: Explanation of how regression derives a causal estimate

Much confusion stems from a lack of detailed comprehension surrounding regression modelling. We may be so familiar with the application of regression yet perhaps unfamiliar with the mechanisms operating from a theoretical perspective. For instance, there is often confusion surrounding the issue that within individuals over a certain age, it is assumed that a person’s height is constant. Within the regression analysis, however, it is not (just) within-person contrasts that are made – no one person is being experimented upon to explore the consequences of their changes in weight (or height) – instead, between person contrasts are being made where both height and weight differ between individuals. Thus, when using regression (informed by a DAG) to estimate counterfactuals (i.e., what if a person had a different height, type of question), causal inference methodology takes advantage of individual differences and similarities in relation to all confounders that have been controlled for.

## Supplementary Section 4: Rationale for using two height variables

A directed acyclic graph (DAG) encodes the established or hypothesised mechanism by which data are generated [2] and then informs which confounders must be controlled for. In this study, the DAG includes both the self-reported baseline height (which may be subject to biased self-reporting) and the measured (follow-up) height that is measured by the research team. There were thus two distinct measures for height. Despite the assumption that there was no actual change in height, the two measurements were not in perfect agreement, indicating a potential bias in the self-reported height. The data generating process for the height variables is two-fold: either recorded by researchers or self-reported, each a distinct manifestation of the true value of height on each occasion. We assumed that measured height was error-free (as measurement errors would be near zero, hence negligible), while we assumed that self-reported height was a combination of unobserved ‘true’ height plus unobserved self-report ‘error’, which thereby makes self-reported height a fully determined variable.

Height is determined by a variety of genetic and lifestyle factors, as too is weight. Consequently, it may be contended that both height and weight share similar underlying causes. This issue can be resolved by modifying the DAG in a way that removes the direct arrow between height and weight and a latent variable may be introduced to account for the numerous common but unrecorded causes of both variables (including measurement errors, which would make the variables fully determined). Given the absence of these variables and the fact that height stabilises within individuals by the age of 23, whereas weight continues to vary, we simplify the DAG by not adding a latent variable (nor measurement errors for weight) and instead use ‘true’ baseline height as a proxy for all these unobserved variables – which is what we did. While it is possible to consider the more complicated DAG, doing so only diverts attention from the primary role that baseline height acts as a confounder for various downstream focal relationships, and additionally acts as a proxy for unmeasured influences on some downstream variables, such as baseline weight. What is more important, as our sensitivity analyses show, is that we capture confounding measures as accurately as possible.

From the perspective of the post-adolescent, we chose true height as the dominant stable feature at baseline. We also clarify that both baseline height and baseline weight act as proxies for common ancestral factors causing both. Consequently, when observing changes in weight, it temporarily follows baseline height and baseline weight. We therefore infer the arc between true baseline height and baseline weight, as well as their influence on all future variables, without the inclusion of any latent variables (which are irrelevant to the arguments being made).

## Supplementary Section 5: A mathematical illustration of how analysis of change relies solely on follow-up outcome

The argument regarding how weight change is derived solely from follow-up weight is shown by examining the expectations (denoted by $E$). Consider, for instance a dietary exposure $A$, where $A=0$ indicates no additional supplements are consumed and $A=a$ indicates that additional supplements are consumed. If we are interested in the average causal effect of the dietary supplement on *weight change*, denoted $W_{1}-W_{0}$, where $W_{1}$ is follow-up weight and $W_{0}$ is baseline weight, this may be written as:

$$E\left[ W_{1}^{a}-W_{0} \right]-E\left[ W_{1}^{0}-W_{0} \right]=E\left[ W_{1}^{a}-W_{0}-W_{1}^{0}+W_{0} \right]=E\left[ W_{1}^{a} \right]-E\left[ W_{1}^{0} \right]$$

where $W_{1}^{a}$ is the expected follow-up weight for individuals consuming the dietary supplement ($A=a)$, $W_{1}^{0}$ is the expected follow-up weight for individuals *not* consuming the dietary supplement ($A=0)$; this hides all potential confounding (including confounding of uptake of dietary supplements by $W_{0}$).

Providing the dietary supplement does not causally affect baseline weight ($W_{0}$), the analysis of weight change in those consuming the dietary supplement and those not consuming the dietary supplement only depends upon the expectations of follow-up weight. The expression indicates that studying the average causal effect of *weight change* amounts to the same as studying the average causal effect on *follow-up weight* (notwithstanding the hidden confounding). If baseline weight ($W_{0}$) is a causal influence on uptake of dietary supplements, $W_{0}$ is a confounder and must be conditioned on. If there is no such confounding of uptake of dietary supplements by $W_{0}$, adjustment for $W_{0}$ is not necessary.

Interpretation of the causal effect of dietary supplements nevertheless depends upon whether $W_{0}$ is conditioned on or not: no adjustment for $W_{0}$ yields a causal effect on *follow-up weight*, while adjustment for $W_{0}$ yields a causal effect on *weight change*. It is thus the combination of how to isolate the change part of the outcome (achieved by follow-up outcome conditioned on baseline outcome) and what other adjustments for confounding are needed (informed by a DAG containing probabilistic variables) that dictates how to estimate the causal effect of an exposure on outcome *change* via its separate follow-up and baseline measures.

## Supplementary Section 6: Rationale for reporting the results from mathematically equivalent models

The analysis of follow-up weight conditional on baseline weight and the analysis of weight change are mathematically equivalent [3]. In RCTs, the analysis of follow-up weight without conditioning on baseline weight should be very similar (albeit slightly less powered for sufficiently sized studies; [4]) due to the randomisation [5], so there is not the same issue. In observational studies, however, to condition on baseline weight it must be a confounder; if it is a mediator, causal mediation analysis must be used. Thus, because randomisation is not present in an observational setting, the correlation between the exposure and the baseline outcome becomes important and the temporal order of baseline outcome weight with respect to baseline exposure dictates whether it is legitimate to adjust (for baseline weight as a confounder) or not (since baseline weight is a mediator).

Although the two different approaches of statistical machinery provide the same numerical answer, the rationale behind each being adopted is different – the strategy that derives the estimation of a causal effect is informed by external knowledge or theory (e.g., the temporal order of variables) which may be encoded in a DAG. It is the underpinning causal inference theory (using the mathematics of graphical model theory or the mathematics of the potential outcomes framework) that dictates which strategy is reliable in observational settings; both mathematical approaches currently only work for probabilistic variables – hence all constructed variables may lead to inferential biases when causal inquiry is sought.

## Supplementary Table S1: Dataset selection - issues with other datasets that were explored.

| **Dataset** | **Year/s** | **Issue** |
| --- | --- | --- |
| National Health and Nutrition Examination Survey (NHANES) | 2014-2014, 2015-2016, 2017-2018, 2017-March 2020 | Cross-sectional study design |
| Active People survey | 2015-2016 | No height and BMI |
| Active Lives Survey | 2018-2019 | No height and weight |
| Health Survey for England | 2018, 2019 | Cross-sectional study design |
| Millennium Cohort Study | 2018 | Under 18s |
| Behavioral Risk Factor Surveillance System (BRFSS) | 2020 | Cross-sectional study design |
| National Child Measurement Programme (NCMP) | 2013-2014 | Under 18s |
| 1970 British Cohort Study (Age 21) | 1992 | No height and weight |
| 1970 British Cohort Study (Age 26) | 1996 | Height and weight either missing completely or not matching across the measurement units |
| 1970 British Cohort Study (Age 29) | 1999-2000 | Height and weight almost completely missing (99.7%+) |
| 1970 British Cohort Study (Age 34) | 2004-2005 | Height and weight either missing completely or not matching across the measurement units |
| 1970 British Cohort Study (Age 38) | 2008-2009 | No height and weight |
| British Household Panel Survey (Waves 1-18) | 1991-2009 | Except for two waves (14 and 16), there are no height or weight measurements. Huge missingness for height and weight in waves 14 and 16 |
| Next Steps: Sweeps 1-8 | 2004-2016 | Age ranges from 14 to 26. Except for sweep 8, there are no height or weight measurements. In sweep 8, the weight in kilogrammes is missing 76.2%, while the weight in stones and pounds is missing 30.9% |
| Understanding Society: Innovation Panel (Waves 1-13) | 2008-2020 | Height and weight hugely missing with values such as -8 (inapplicable) for waves 1, 2, 3, 4, 5, 6, and 12. No height and weight measurements for waves 7, 8, 9, 10, and 11 |
| National Child Development Study - Age 42 (Sweep 6) | 1999-2000 | Height missing completely, weight missing 99.99% |
| National Child Development Study - Age 46 (Sweep 7) | 2004-2005 | No height and weight |
| National Child Development Study - Age 50 (Sweep 8) | 2008-2009 | Height is missing 93.1% |

## Supplementary Table S2: Distinct role of birth and baseline variables.

| **Birth variables** | **Baseline variables** |
| --- | --- |
| **Birth variables** are distinct from **baseline variables** (at age 23) because their causal impact on the outcome (follow-up weight) is different – **birth variables** temporally precede baseline weight and causal mediation analysis (CMA) is therefore required if asking what their causal effect is on ‘weight change’; without CMA, standard regression will estimate their causal impact on *follow-up weight*, not ‘weight change’. | B**aseline exposure variables** occur after baseline weight, and standard regression will estimate their causal impact on *follow-up weight conditional on baseline weight* (with the latter treated as a confounder) and this thus estimates their causal impact on ‘weight change’ (by implication of the conditioning on baseline weight). |

## Supplementary Table S3: Model results: linear model regression coefficient estimates and 95% confidence intervals (CIs) for each model.

| **Exposure** | **Adjustment Set** | **Reason for Adjustment** | **Outcome Model** | **Estimate (95% CI)** |
| --- | --- | --- | --- | --- |
| Sex | Ethnicity | Competing Exposure | Follow-up Weight | 14.85 (14.30, 15.40) |
|  |  |  | Weight Change | 0.495 (0.129, 0.861) |
|  |  |  | Relative Weight Change | -1.425 (-1.975, -0.875) |
|  |  |  | Follow-up BMI | 1.051 (0.868, 1.235) |
|  |  |  | BMI Change | 0.146 (0.014, 0.278) |
|  |  |  | Relative BMI Change | 0.370 (-0.202, 0.942) |
| Ethnicity | Sex | Competing Exposure | Follow-up Weight | -3.088 (-5.121, -1.054) |
|  |  |  | Weight Change | 1.075 (-0.273, 2.423) |
|  |  |  | Relative Weight Change | 2.408 (0.382, 4.435) |
|  |  |  | Follow-up BMI | -0.002 (-0.679, 0.674) |
|  |  |  | BMI Change | 0.534 (0.047, 1.021) |
|  |  |  | Relative BMI Change | 2.346 (0.240, 4.453) |
| Reported Height | Ethnicity, Sex | Confounders | Follow-up Weight | 0.668 (0.630, 0.707) |
|  |  |  | Weight Change | 0.039 (0.012, 0.066) |
|  |  |  | Relative Weight Change | -0.044 (-0.085, -0.004) |
|  |  |  | Follow-up BMI | -0.030 (-0.044, -0.017) |
|  |  |  | BMI Change | 0.022 (0.012, 0.031) |
|  |  |  | Relative BMI Change | 0.118 (0.076, 0.161) |
| Baseline Weight | Ethnicity, Sex, Reported Height | Confounders | Follow-up Weight | 1.059 (1.038, 1.080) |
|  |  |  | Weight Change | 0.059 (0.038, 0.080) |
|  |  |  | Relative Weight Change | -0.058 (-0.090, -0.026) |
|  |  |  | Follow-up BMI | 0.359 (0.351, 0.366) |
|  |  |  | BMI Change | 0.012 (0.004, 0.020) |
|  |  |  | Relative BMI Change | -0.104 (-0.137, -0.070) |
| Economic Status | Ethnicity, Sex, Reported Height, Baseline Weight, Economic Status | Confounders | Follow-up Weight | 0.184 (-0.278, 0.645) |
|  |  |  | Weight Change | 0.184 (-0.278, 0.645) |
|  |  |  | Relative Weight Change | 0.500 (-0.195, 1.195) |
|  |  |  | Follow-up BMI | 0.013 (-0.155, 0.182) |
|  |  |  | BMI Change | -0.015 (-0.182, 0.152) |
|  |  |  | Relative BMI Change | 0.081 (-0.639, 0.801) |
| Malaise Score | Ethnicity, Sex, Reported Height, Baseline Weight, Economic Status | Confounders | Follow-up Weight | 0.131 (-0.594, 0.857) |
|  |  |  | Weight Change | 0.131 (-0.594, 0.857) |
|  |  |  | Relative Weight Change | 0.091 (-1.001, 1.183) |
|  |  |  | Follow-up BMI | 0.119 (-0.146, 0.384) |
|  |  |  | BMI Change | 0.150 (-0.112, 0.412) |
|  |  |  | Relative BMI Change | 0.388 (-0.744, 1.520) |
|  | | | | |

## Supplementary Table S4: Linear model coefficient estimates and 95% confidence intervals (CIs) for models that are mathematically equivalent.

| **Exposure** | **Adjustment Set** | **Outcome Model** | **Estimate (95% CI)** |
| --- | --- | --- | --- |
| Ethnicity | Sex, Reported Height, Baseline Weight | Follow-up Weight | 1.327 (-0.023, 2.676) |
|  |  | Weight Change | 1.327 (-0.023, 2.676) |
|  | Sex, Baseline BMI | Follow-up BMI | 0.552 (0.065, 1.039) |
|  |  | BMI Change | 0.552 (0.065, 1.039) |
| Economic Status | Ethnicity, Sex, Reported Height, Baseline Weight, Economic Status | Follow-up Weight | 0.184 (-0.278, 0.645) |
|  |  | Weight Change | 0.184 (-0.278, 0.645) |
|  | Ethnicity, Sex, Baseline BMI, Economic Status | Follow-up BMI | -0.056 (-0.222, 0.111) |
|  |  | BMI Change | -0.056 (-0.222, 0.111) |
| Malaise Score | Ethnicity, Sex, Reported Height, Baseline Weight, Economic Status | Follow-up Weight | 0.131 (-0.594, 0.857) |
|  |  | Weight Change | 0.131 (-0.594, 0.857) |
|  | Ethnicity, Sex, Baseline BMI, Economic Status | Follow-up BMI | 0.174 (-0.088, 0.437) |
|  |  | BMI Change | 0.174 (-0.088, 0.437) |
| Outcome follow-up conditioned on outcome baseline yields identical effect estimates to outcome change-score conditioned on outcome baseline. From a theoretical perspective, the only viable causal inference estimate is that for non-composite outcomes (i.e., follow-up weight) informed by the DAG, since the theory underpinning DAGs is viable only for non-composite variables (outcomes and exposures). | | | |

Table S4 shows that models for follow-up weight and weight change-score yield equivalent estimates providing both condition on baseline weight; similarly, models for follow-up BMI and BMI change-score yield equivalent estimates providing both condition on baseline BMI. Models for weight outcomes differ considerably from models for BMI outcomes – the only viable causal estimates are those from models with non-composite outcomes conditioned on an adjustment set informed by a DAG in which neither the exposure nor the outcome is composite (else the underpinning graphical model theory is violated).

To examine the causal effect of an exposure on the *change* in an outcome, only models with the outcome follow-up conditioned on the outcome baseline correctly isolates the ‘*change*’ component; even then, only models where the outcome (and exposure) is not composite can the appropriate adjustment set be informed by the DAG.

## Supplementary Figure S1: A flow chart of sample selection from the National Child Development Study (NCDS) cohort surveys.

**^✝^**It is important to recognise that the dataset used in this study included only the 9,223 individuals who remained in the study by age 33 years, whereas the number of individuals at age 23 years was 12,537 – the original sample thus suffered 26.4% loss to follow-up for the key variables considered.

This level of dropout is substantial and could create large biases in the estimates sought if not addressed – possibly through multiple imputation [8], inverse probability weighting [9], or both [10], and perhaps combined with full information maximum likelihood (FIML) [11]. It is however beyond the scope of this article to illustrate such procedures.

Since the resulting biases incurred by not addressing loss-to-follow-up might be as large or larger than composite variable bias, all reported causal effect estimates in our study may be biased. Our article thus seeks only to be illustrative of the issues surrounding composite outcomes and should not be viewed as a serious endeavour to obtain population-specific causal effects estimates.

## Supplementary Figure S2: Data summary statistics: frequency, distribution plot, number and percentage of valid and missing observations in the dataset analysed.

## Supplementary Figure S3: Directed Acyclic Graph (DAG) for the estimated causal effect of economic status (exposure) on follow-up weight (outcome) adjusting for confounders ethnicity, sex, baseline height (using self-reported height as a proxy for true height), and baseline weight (to ensure the resulting estimate pertains to the outcome ‘change’).

## Supplementary Figure S4: A comparison of the results for different weight outcomes for different exposure variables of interest with measured height used instead of the self-reported height – the dotted vertical line separates the birth variables from the baseline (age 23) variables (see Table S3). Results differ slightly from those using self-reported height, revealing the importance of height being accurately measured.^[[1]](#footnote-1)^

## Supplementary Figure S5: Comparing results with (left) and without (right) adjustment for baseline height and baseline weight for exposures economic status and malaise score.

# References

1. VanderWeele TJ. Explanation in Causal Inference: Methods for Mediation and Interaction. New York: Oxford University Press; 2015.

2. Byeon S, Lee W. Directed acyclic graphs for clinical research: a tutorial. Journal of Minimally Invasive Surgery. 2023;26: 97.

3. O’Connell NS, Dai L, Jiang Y, Speiser JL, Ward R, Wei W, et al. Methods for analysis of pre-post data in clinical research: a comparison of five common methods. Journal of biometrics & biostatistics. 2017;8: 1.

4. Tu YK, Blance A, Clerehugh V, Gilthorpe MS. Statistical power for analyses of changes in randomized controlled trials. JDentRes. 2005;84: 283–287.

5. Greenland S, Mansournia MA. Limitations of individual causal models, causal graphs, and ignorability assumptions, as illustrated by random confounding and design unfaithfulness. European journal of epidemiology. 2015;30: 1101–1110.

6. Suttorp MM, Siegerink B, Jager KJ, Zoccali C, Dekker FW. Graphical presentation of confounding in directed acyclic graphs. Nephrology Dialysis Transplantation. 2015;30: 1418–1423. doi:10.1093/ndt/gfu325

7. Ellison GTH. Introducing causal inference to the medical curriculum using temporal logic to draw directed acyclic graphs. medRxiv; 2020. p. 2020.08.02.20166900. doi:10.1101/2020.08.02.20166900

8. Carpenter J, Kenward M. Multiple Imputation and its Application. London: John Wiley & Sons; 2012.

9. Seaman SR, White IR. Review of inverse probability weighting for dealing with missing data. StatMethods MedRes. 2013;22: 278–295.

10. Seaman SR, White IR, Copas AJ, Li L. Combining multiple imputation and inverse-probability weighting. Biometrics. 2012;68: 129–137.

11. Enders CK. The performance of the full information maximum likelihood estimator in multiple regression models with missing data. Educational and Psychological Measurement. 2001;61: 713–740.

1. The effect of ethnicity and sex differences on follow-up weight were ten-fold greater than the coefficients shown in the plot and were rescaled to make the plot more readable [↑](#footnote-ref-1)
