# Supplementary figures and images for: Composite variable bias: causal analysis of weight outcomes
Source: Int J Obes (Lond). 2025 Mar 8;49(6):1043–50. doi: 10.1038/s41366-025-01732-6 (PMC12158777; doi:10.1038/s41366-025-01732-6)

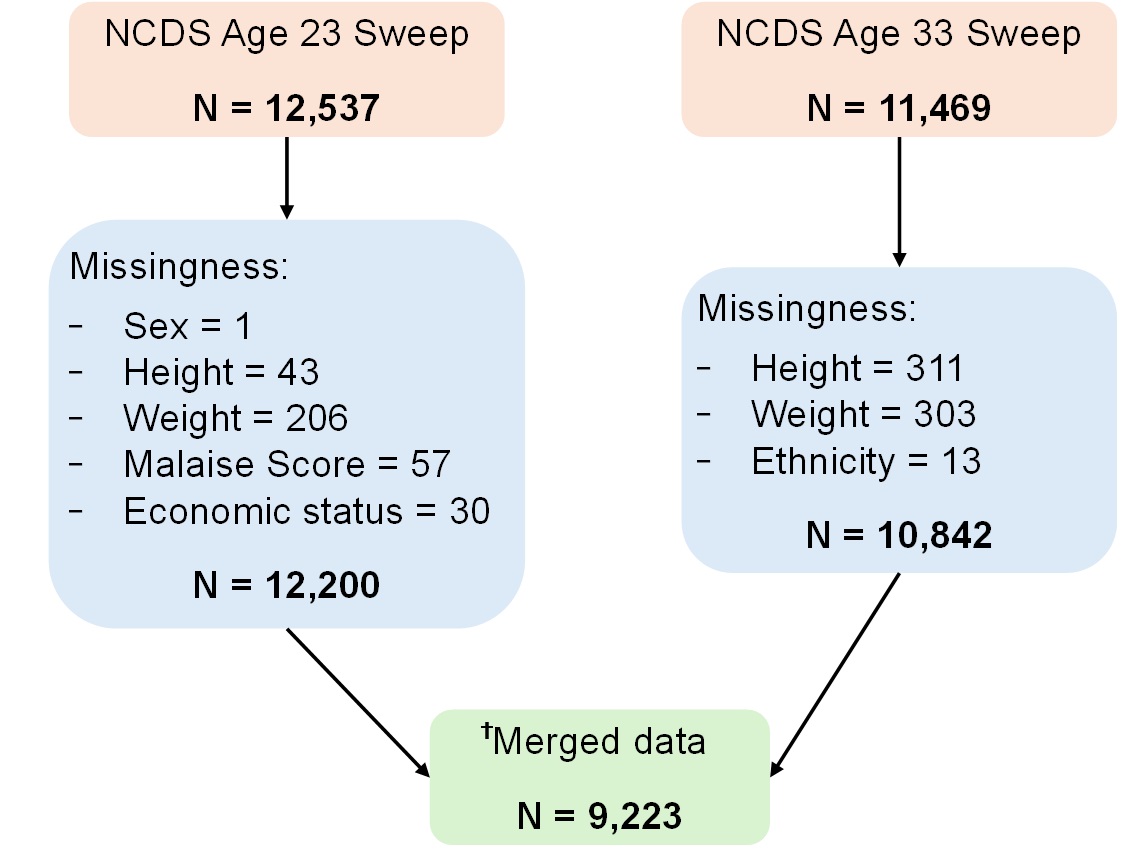

Supplement: Supplementary file 2 — Supplementary Figure S1 [file 41366_2025_1732_MOESM2_ESM.jpg]

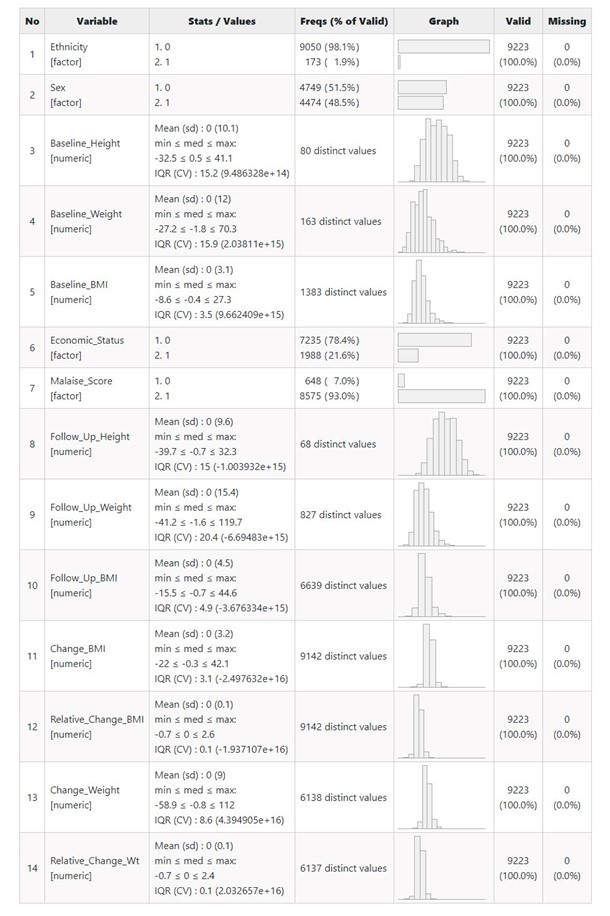

Supplement: Supplementary file 3 — Supplementary Figure S2 [file 41366_2025_1732_MOESM3_ESM.jpg]

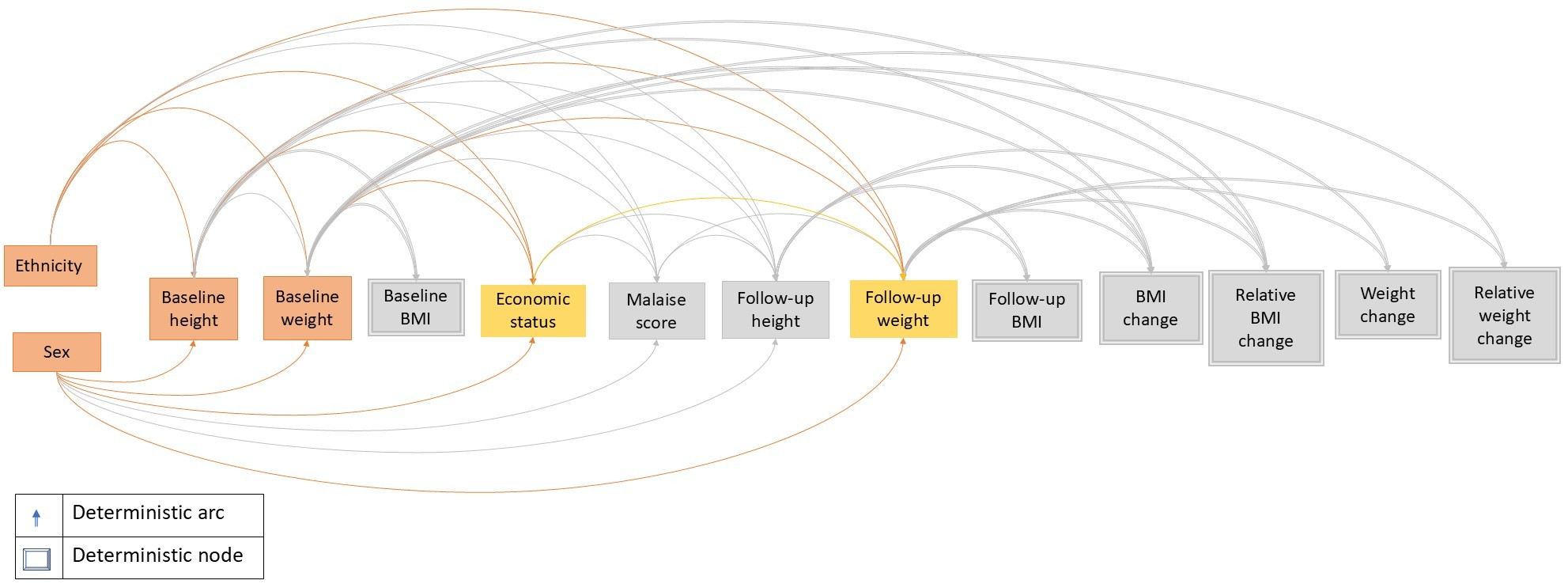

Supplement: Supplementary file 4 — Supplementary Figure S3 [file 41366_2025_1732_MOESM4_ESM.jpg]

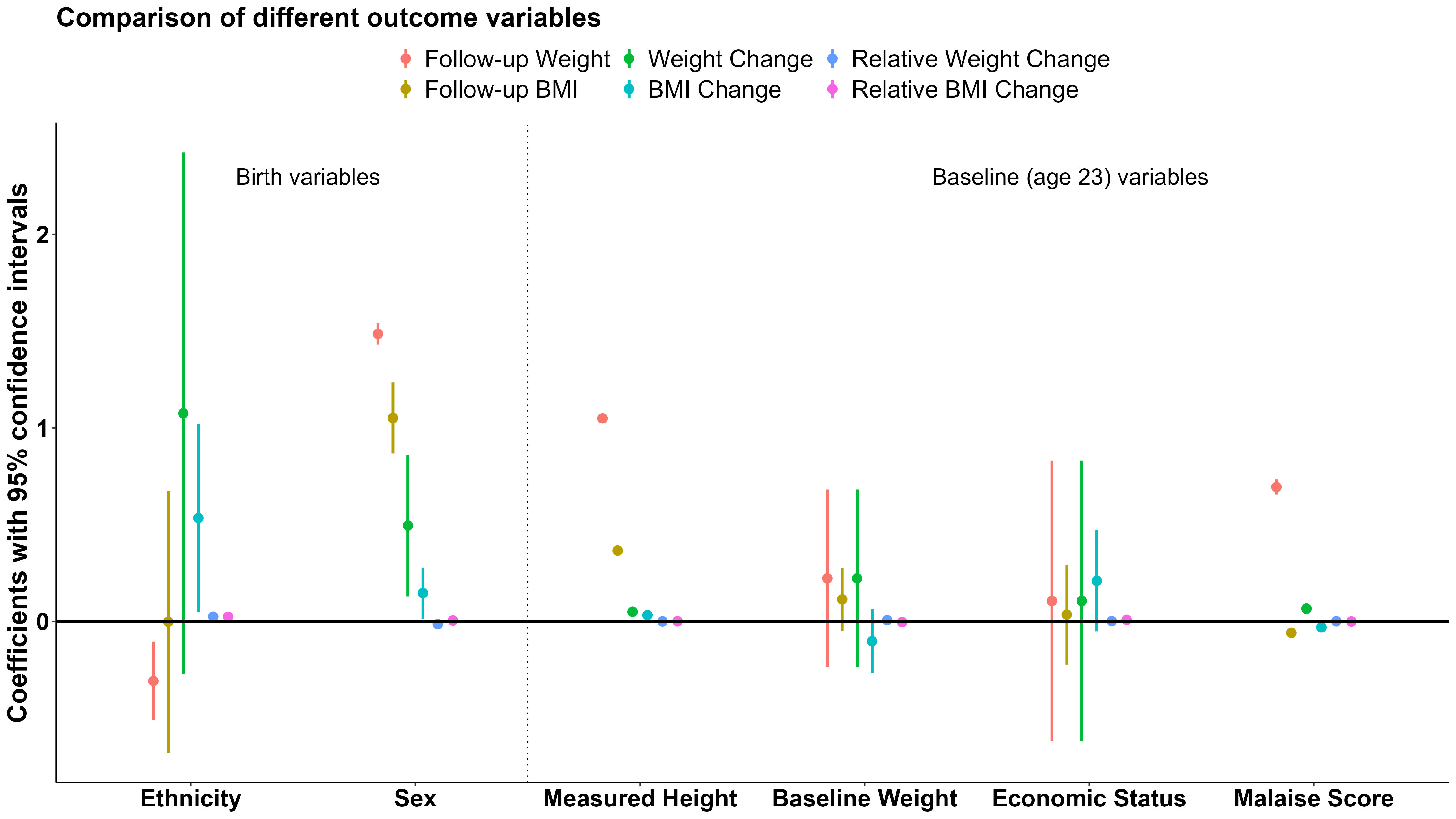

Supplement: Supplementary file 5 — Supplementary Figure S4 [file 41366_2025_1732_MOESM5_ESM.jpg]

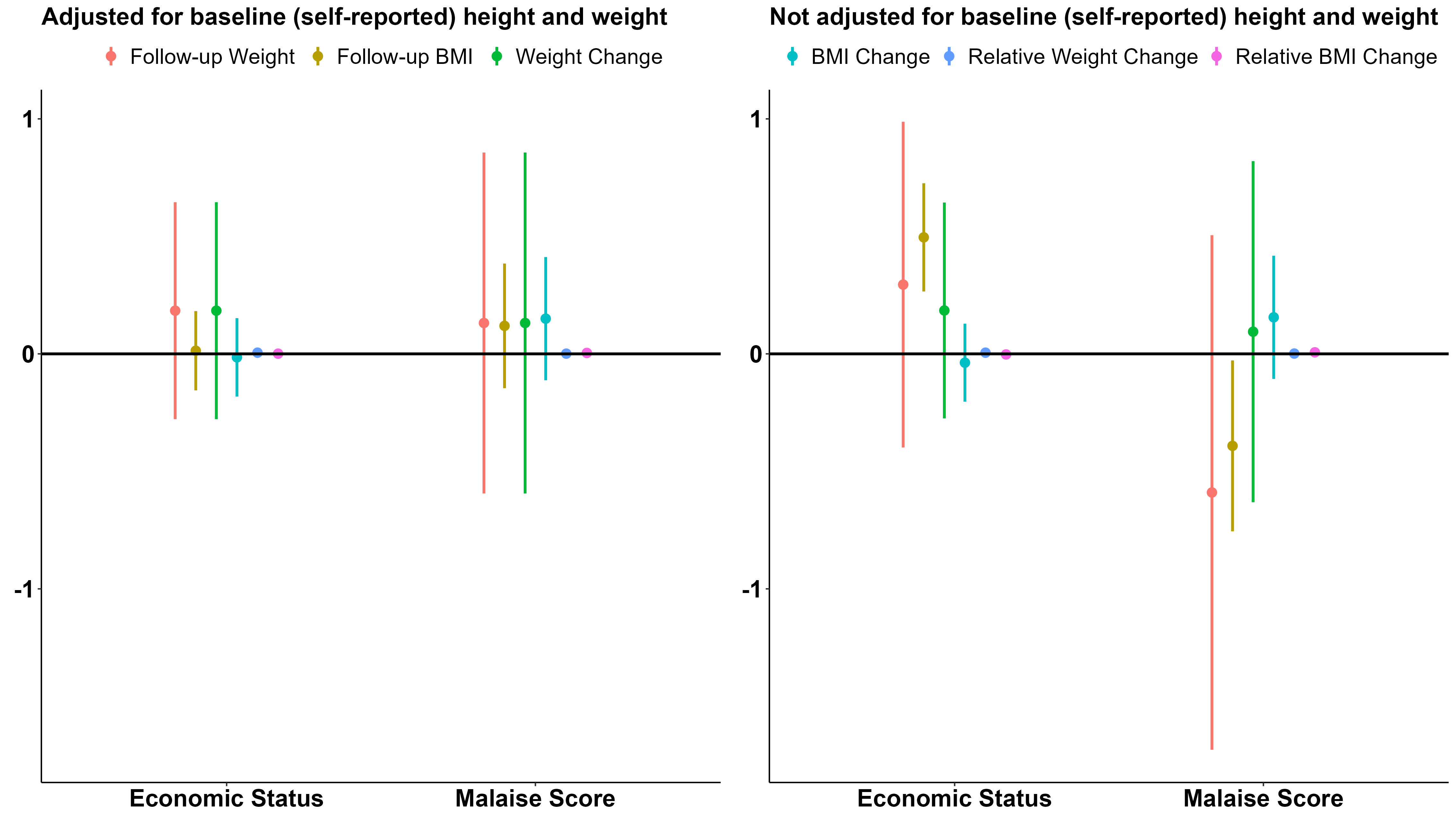

Supplement: Supplementary file 6 — Supplementary Figure S5 [file 41366_2025_1732_MOESM6_ESM.jpg]
